# Heat shock protein 70 protects mouse against post-infection irritable bowel syndrome via up-regulating intestinal γδ T cell’s Th17 response

**DOI:** 10.1186/s13578-018-0237-z

**Published:** 2018-06-05

**Authors:** Zhoutao He, Xiaoning Sun, Zhichao Ma, Jiao Fu, Baili Huang, Fujin Liu, Yi Chen, Taozhi Deng, Xiangyang Han, Deming Sun, Cheng Lan

**Affiliations:** 1 Department of Gastroenterology, Hainan General Hospital, Xiu Hua Road 19th, Xiu Ying District, Haikou, 570311 Hainan Province China; 2 Medical Research Center, Hainan General Hospital, Haikou, 570311 Hainan Province China; 3 Department of Phathology, Hainan General Hospital, Haikou, 570311 Hainan Province China; 4 Doheny Eye Institute, Department of Ophthalmology, David Geffen School of Medicine, University of California Los Angeles, Los Angeles, CA 90033 USA

**Keywords:** Heat shock protein 70, γδ T cells, Post-infectious irritable bowel syndrome, Th17 response, Mice

## Abstract

**Background:**

This study investigated the role of HSP70 in modulating intestinal γδ T cells’ Th17 response in *Trichinella spiralis*-induced PI-IBS mice model.

**Methods:**

The intestinal HSP70’s expression and mRNA level were measured by Western blot and RT-PCR. The intestinal γδ T cell’s morphological changes were analyzed using immunofluorescence staining and confocal laser scanning microscope. The pro-inflammatory cytokines’ level was detected by ELISA. The isolated and purified γδ T cells were pre-incubated with HSP70 and their functions including proliferation, apoptosis, activation and production of IL-17 were also detected.

**Results:**

Heat treatment augmented intestinal HSP70 expression and alleviated the clinical presentations in PI-IBS mice. Meanwhile, intestinal γδ T cells and local IL-17 level were increased by pre-induction of HSP70. HSP70 promoted the proliferation of PI-IBS mice’s intestinal γδ T cells, inhibited the apoptosis and stimulated these cells to secret IL-17 rather than IFN-γ.

**Conclusion:**

Our results suggest that HSP70 plays a protective role via up-regulating intestinal γδ T cell’s Th17 response in PI-IBS mice.

## Background

As a kind of clinical syndrome characterized by abdominal pain, discomfort and bloating accompanied with abnormal defecation, the precise patho-physiological mechanism of irritable bowel syndrome (IBS) remains unclear [1–3]. During the last two decades, abundant clinical and experimental research focused on the role of infection and inflammation in the pathogenesis of IBS, called as post-infectious irritable bowel syndrome (PI-IBS) [4–6].

The pathophysiology of PI-IBS is still not fully understood, but low-grade inflammation and chronic alteration of the immune system at the molecular level have been shown to be associated with mucosal secretory function, smooth muscle and enteric nervous fibers [7–9]. In particular, an imbalance of pro- and anti-inflammatory cytokines is found, which may play a key role in the local intestinal inflammation. Therefore, experimental infection with the parasite Trichinella has been widely used to establish models for detecting the pathogenesis of intestinal dysfunction [10, 11]. Infection by *Trichinella spiralis* larvae induced changes in visceral sensitivity, alterations of intestinal smooth muscle function, and altered secretion. These abnormalities persisted after animals recovered from infection, suggesting that this is a suitable model of PI-IBS. Activated immune cells continue to release various cytokines after an acute intestinal infection [12], for example, T-helper (Th) cells produce interferon (IFN)-γ and interleukin (IL)-1β to promote the inflammatory response; T-regulatory cells release IL-10 to prevent autoimmunity; in contrast, IL-17, which is produced by Th17 cells, can induce autoimmunity [12]. These cytokines may alter the physiology and immunity of the host gut to cause symptoms of PI-IBS. The HSP70 family of heat shock proteins consists of molecular chaperones of approximately 70 kDa in size that serve critical roles in survival function in the cell. Recently, it was reported that heat shock protein 70 (HSP70) has a unique capability of regulating the protein misfolding, aggregation and serves critical roles in some diseases [13, 14]. However, the effects of HSP70 on PI-IBS have not been reported. γδ T cells were proved to participate in inflammatory and autoimmune disorders [15–17]. Thus, the current study aimed to investigate whether HSP70 regulated the γδ T cells’ phenotype and function during PI-IBS.

## Methods

### Animals and study design

96 female 57BL/6 mice (6–8 weeks old and 13–15 g weigh) were purchased from Medical Animal Center, the Hainan medical College. All animals were housed in sterile, pathogen-free, temperature controlled facility on normal 12-h light/dark cycle, and standard diet and water were provided ad libitum. The animals were randomly assigned into four groups: control group, PI-IBS group, Heat + PI-IBS group and Heat group (n = 24 in each group). In each group, six mice were sacrificed for the detection of the intestinal HSP70 protein level, and six mice for HSP70 mRNA level. six mice were used for the isolation, purification and culture of the intestinal γδ T cells. The other six mice were examined for the visceral hypersensitivity and the intestinal motility.

### Modeling of PI-IBS

The mice were infected with *T. spiralis* (Lanzhou Animal Medical Institute, Lanzhou, China) as described previously [18]. Briefly, the parasite larvae were separated from Sprague–Dawley rats 60 days after infection of *T. spiralis*’ cyst by digestion with 1.5% gastric pepsin (Invitrogen Co., CA, USA). The mice were fed with the larvae in 0.2 ml saline (300 larvae per mouse). The animals in the control group were fed with only 0.9% saline.

### Histopathological study

The animals were sacrificed at the 8th weeks after infection and their ileum tissue fixed within 10% formalin in PBS at 4 °C, dehydrated in a graded series of ethanol, and then embedded in paraffin wax. The tissues were sectioned at 5 μm thick and mounted on slides. The slides were dewaxed, hydrated, and then stained with hematoxylin–eosin (HE). The HE staining was further used for evaluating inflammatory score basing previous scoring system [19].

### Abdominal withdrawal reflex (AWR)

AWR was performed to evaluate the visceral hypersensitivity [20]. The anesthetized animals were inserted via their anus with air chamber and catheter. The air chamber was distended at volume of 0.25/0.35/0.5/0.65 ml × l5 min × 3 times. Between each distending time, the animals were permitted to have a rest for 30 s. The AWR scoring standard: when stimulated, the animals are in stable mood, 0 point; if the animals are in unstable mood, twisting their heads once in a while, 1 point; slightly contracting their abdomen and back muscles, 2 points; intensively contracting their abdomen muscles and uplifting the abdomen from the ground, 3 points; intensively contracting abdomen muscles, bowing abdomen and uplifting the abdomen and perineum, 4 points.

### Colon Transportation Test (CTT)

CTT was performed to evaluate the status of the intestinal motility. After filled into stomach with 0.4 ml active carbon, the first black stool time was recorded. The total stool within 8 h was collected and evaluated by Bristol stool grade [21]: normal shaped stool, 1 point; soft or deformed stool, 2 points; water-like stool, 3 points.

### Preinduction of HSP70

Expression of HSP70 was induced by heat treatment according to previous report [22]. The mice were anesthetized with sodium pentobarbital (50 mg/kg). Rectal temperature was monitored with a thermistor inserted into the rectum in a baking oven with constant temperature 50 centigrade. After the body temperature was maintained at 41 °C form 20 min, the mice were return to their cages at room temperature and allowed water and food as libitum. Non-heated mice were only anesthetized but received no hyperthermic stress.

### Determination of HSP70

#### Western blot

HSP70 protein level was measured by Western blot (Wuhan Boster Coporation, Wuhan, China). Briefly, the ileum tissue sample was grinded and cracked with RIPA. The homogenate was centrifuged for 30 min. The protein concentration in the supernatants was measured by Bradford Assay. 40 µg tissue sample was separated by SDS page gel electrophoresis and transferred to the PVDF membrane. The membrane was blotted with TBST for 1 h, then was added with anti-mouse HSP70 multiple clone antibodies (1:1000) (ab2787, abcam, US) and rabbit anti-mouse—actin multiple clone antibodies (1:1000) (ab8226, abcam, US) at 4 °C for 12 h. One day later, the membrane was washed in TBST and autographied by ECL chemiluminescent assay. The gray-scale value was detected by enhanced chemiluminescence in dark room. The gray-scale value of Hsp70/-actin represented the relative expression level of HSP70.

#### Real-time quantitative PCR (RT-PCR)

HSP70 mRNA level was measured by RT-PCR. Total RNA was isolated from the terminal ileum tissue with Trizol liquid and treated with DNAase I. Primer was designed according to mouse gene sequence. β-actin worked as an internal control.

HSP70 gene primer sequence (5′-3′):

- F: GAAGGTGCTGGACAAGTGC,
- R: GCCAGCAGAGGCCTCTAATC.

β-actin gene primer sequence (5′-3′):

- F: AGGCTGTGCTGTCCCTGTATG,
- R: GAGGTCTTTACGGATGTCAACG.

RT-PCR was operated according to following protocol: 1. Pre-denaturation program (5 min at 94 °C); 2. Denaturation program (1 min at 94 °C); 3. Amplification and qualification program, repeated 30 cycles (50 s at 57 °C, 20 s at 60 °C); 4. Prolonging program (7 min at 72 °C). The relative expression was expressed as a ratio of the target gene to the control gene.

### Morphological analysis

The ultrathin frozen section of the ileum tissue was treated by immunofluorescence histochemical staining. The primary antibody was florescence- labeled rat anti-mouse anti-TCR antibody (GL3 clone). The tissue sections were scanned under Laser scanning confocal microscope. The intensity of fluorescence was calculated automatically using the image analysis software.

### Isolation and purification of γδ T cells

The isolation and purification of γδ T cells were conducted as described by Cheng et al. [23]. Briefly, total T cells were isolated with collagenase D digestion method (Roche, Basel, Switzerland) followed by centrifuging with lymphocyte separation medium Ficoll (Sigma-Aldrich, ST. Louis, MO, USA). The total T cells were stained with FITC conjugated anti-δ TCR mAb (100 μl antibodies per 108 cells, incubated at 4 °C for 30 min), followed by microbeads conjugated anti-FITC mAb staining (100 μl antibodies per 108 cells, incubated at 4 °C for 30 min). MACS was used to select the positive cells for two times. FITC conjugated anti-TCR mAb and microbeads conjugated anti-FITC mAb staining were used to clear the miscellaneous γδ T cells. The living cells remain up to more than 95%.

### Determination of γδ T cells’ function in vitro

#### Proliferation

CFSE proliferation assay was performed to measure the proliferation of γδ T cells. The purified γδ T cells were pre-incubated with HSP70 or not. Briefly, 4–8 × 10^6^ γδ T cells were stained with 3.5 μM CFSE (Sigma-Aldrich, ST. Louis, MO, USA) for 4 min at RT. 1.5 × 10^5^ CFSE-labeled γδ T cells/ml were incubated in a 96-well plates for 48 h with Hsp70 10 ng/ml, 1.5 × 10^5^ spleen cells irradiated with γ ray (as antigen presenting cells), *T. spiralis* excretory–secretory-antigen 10 μg/ml (Sigma-Aldrich, ST. Louis, MO, USA), the total volume per well was 200 μl. FACS analysis was performed to measure the CFSE signal/cell with a reduction of intensity as a marker of proliferation.

#### Activation

γδ T cells were stained with PE conjugated anti-CD69 mAb and anti-CD62L mAb (100 μl antibodies per 1 × 10^8^ cells, incubated with IL-23 and TLR4 at 4 °C for 30 min). FACS was used to determine the expression of CD69 and CD62L on γδ T cells [24].

#### Apoptosis

The purified γδ T cells were pre-incubated with HSP70 and their apoptosis was detected with Annexin V-FITC/PI Apoptosis Detection Kit (Sigma-Aldrich, St. Louis., MO, USA) in accordance with the manufacture’s instruction.

### Determination of proinflammatory cytokines

The tissue sample was ultrasonically shivered and centrifuged at 4 °C for 15 min. The concentration of IL-17 in the supernatants from the intestinal tissue sample and the culture of γδ T cells were measured by ELISA.

### Statistics analysis

Data were analyzed using Student’s *t* test (SPSS 17.0 software) and AVNOVA test. Data were expressed as the mean ± standard error. p < 0.05 was considered as statistically significant difference.

### Ethical considerations

The experiment was carried out in accordance with the Chinese guidelines for animal welfare. Experimental protocol was approved by the Animal Care and Use Committee of Hainan Province.

## Results

### AWR scores

The anesthetized animals were inserted via their anus with air chamber and catheter. The air chamber was distended at volume of 0.25/0.35/0.5/0.65 ml × l5 min × 3 times. The AWR score is from 0 to 4 according with the animals’ mood when stimulated. As a result, between 0.35 ml and 0.5 ml, PI-IBS mouse show higher AWR score than control group (p < 0.05). Heat pretreatment significantly decreased the AWR score in PI-IBS mouse (p < 0.05) but not to the normal level. These results show that hear pretreatment improved the visceral hypersensitivity in PI-IBS mouse (Table 1).

**Table 1:** AWR score in heat pretreated PI-IBS mice

Distending air volume (ml)	AWR score			
	0.25	0.35	0.5	0.65
Control (n = 7)	0.00 ± 0.00	1.42 ± 0.53	2.67 ± 0.53	3.57 ± 0.51
PI-IBS (n = 7)	0.00 ± 0.00	2.57 ± 0.52	3.57 ± 0.50	3.72 ± 0.49
Heat + PI-IBS (n = 7)	0.00 ± 0.00	2.14 ± 0.39^a^	2.74 ± 0.49^b^	3.76 ± 0.38
Heat (n = 7)	0.00 ± 0.00	1.24 ± 0.67	1.86 ± 0.69	3.61 ± 0.48

^a^ Compared with the PI-IBS group, *p* < 0.05

^b^ Compared with the PI-IBS group, *p* < 0.05

### Colon Transportation Test

CTT was performed to evaluate the status of the intestinal motility. The total stool within 8 h was collected and evaluated by Bristol stool grade 1–3. As shown in (Table 2), the first black stool time in PI-IBS mouse obviously shorten, which was partially prolonged in heat-pretreated PI-IBS mouse (p < 0.05). Bristol stool grade show the grade in PI-IBS mouse was obviously increased, which was partially decreased in heat-pretreated PI-IBS mouse (p < 0.05). These results show that heat pretreatment improved the intestinal motility in PI-IBS mouse (Table 2).

**Table 2:** Changes of the intestinal mobility in heat pretreated PI-IBS mice

Group	First black stool time (minutes)	Bristol stool grade
Control (n = 7)	108.29 ± 13.06	0.97 ± 0.11
PI-IBS (n = 7)	68.97 ± 5.96	2.86 ± 0.44
Heat + PI-IBS (n = 7)	88.74 ± 5.90^a^	2.17 ± 0.18^b^
Heat (n = 7)	102.43 ± 12.18	1.15 ± 0.21

^a^ Compared with the PI-IBS group, *p* < 0.05

^b^ Compared with the PI-IBS group, *p* < 0.05

### Effect of heat treatment on expression of HSP70 in PI-IBS mice

**Fig. 1:**
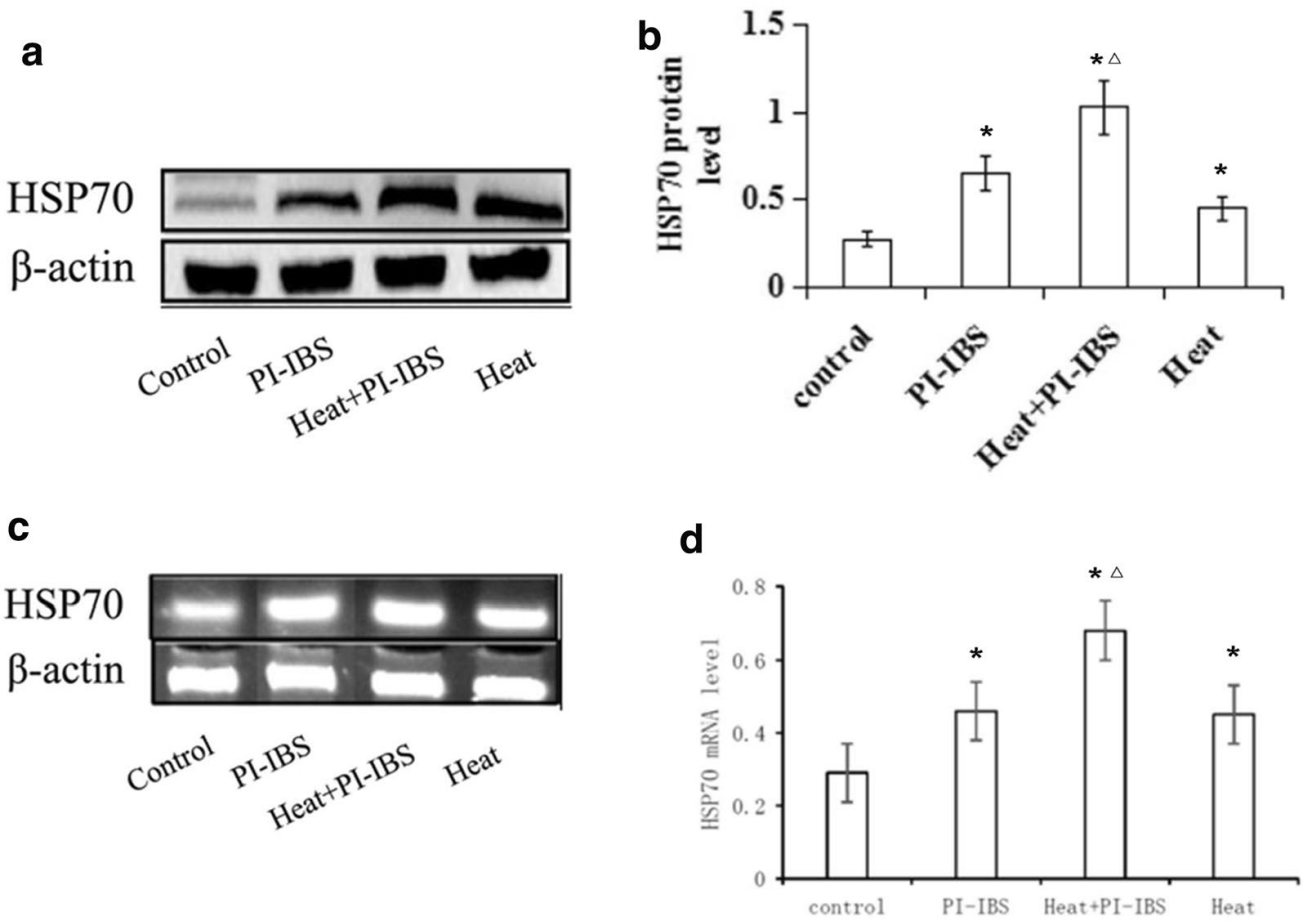
Protein and mRNA expression of HSP 70. **a**, **b** Expression of HSP 70 detected by Western blot; **c**, **d** Expression of HSP 70 detected by RT-PCR. *p < 0.05 compared with control group; △, p < 0.05 compared with both HSP70 group and IP-IBS group

Both of the protein and mRNA expression of HSP70 was significantly higher in HSP group than that in control group (p < 0.05), suggesting that heat treatment upregulated HSP70 expression. Meanwhile, *T. spiralis* infection in IP-IBS group has also markedly upregulated HSP70 expression (p < 0.05). Interestingly, when the *T. spiralis* infected mice were also given heat treatment, the expression of HSP70 became even higher, which was significantly higher than both HSP70 group and IP-IBS group (p < 0.05) (Fig. 1).

### Intestinal γδ T cells and IL-17 level were increased in heat-treated PI-IBS mice

**Fig. 2:**
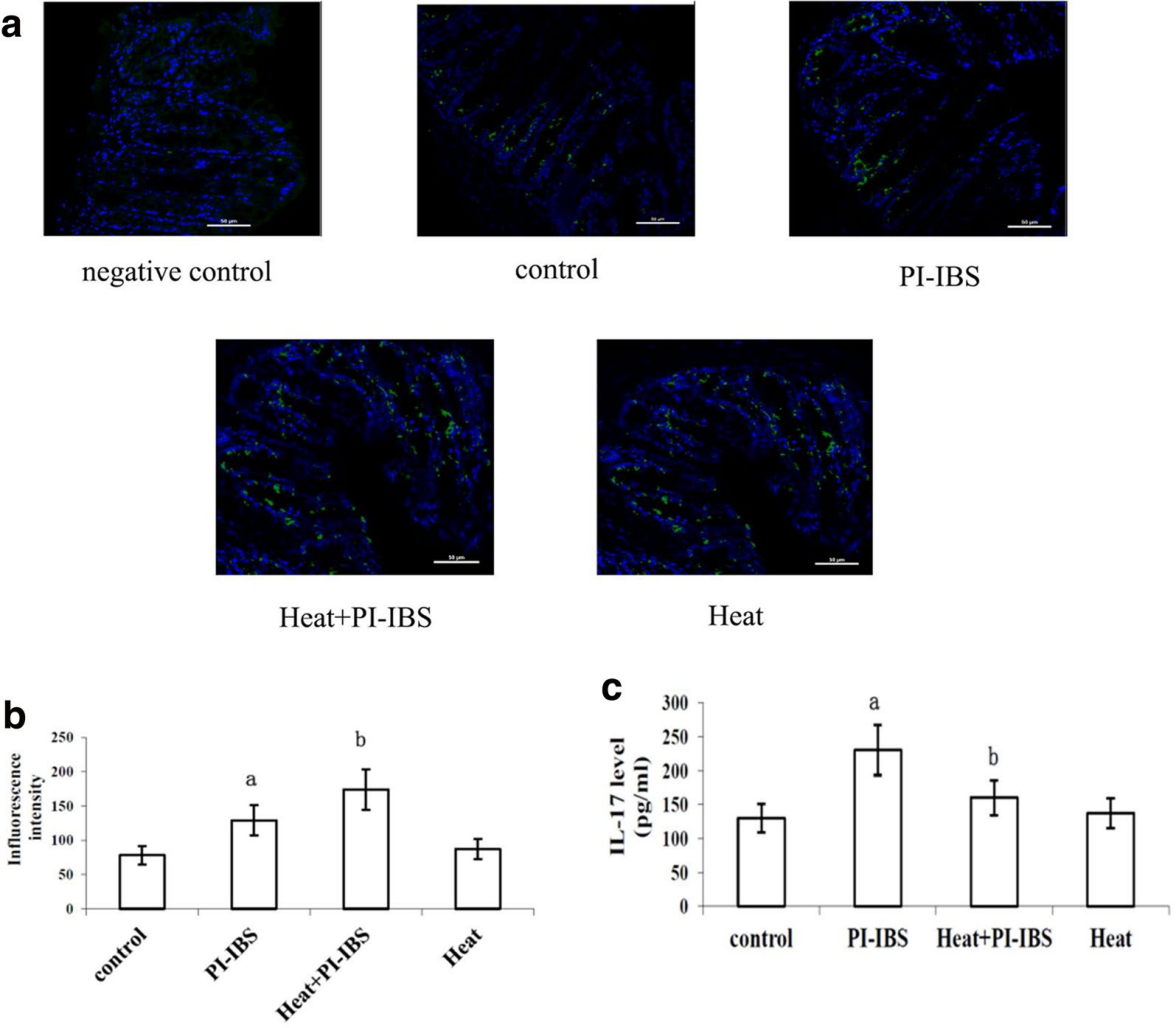
γδ T cells and IL-17 level in PI-IBS mice. **a** Stained γδ T cells of different groups were observed under confocal microscope; **b** Fluorescence intensity of γδ T cells in different groups; **c** IL-17 levels of different groups

Stained γδ T cells of different groups were observed under confocal microscope (Fig. 2a). The fluorescence intensity of the γδ T cells was detected. It was shown that the fluorescence intensity was significantly higher in PI-IBS group and HSP group compared with control group (p < 0.05). However, after the heat treatment on PI-IBS mice, the fluorescence intensity became the highest, which was similar with the results of HSP70 expression (Fig. 2b).

IL-17 of the intestinal tissue sample was then measured by ELISA. The expression of IL-17 was significantly increased in PI-IBS group compared with control group, while it was significant decreased by heat treatment (p < 0.05) (Fig. 2c).

### Heat treatment improved the inflammatory score in PI-IBS mice

**Fig. 3:**
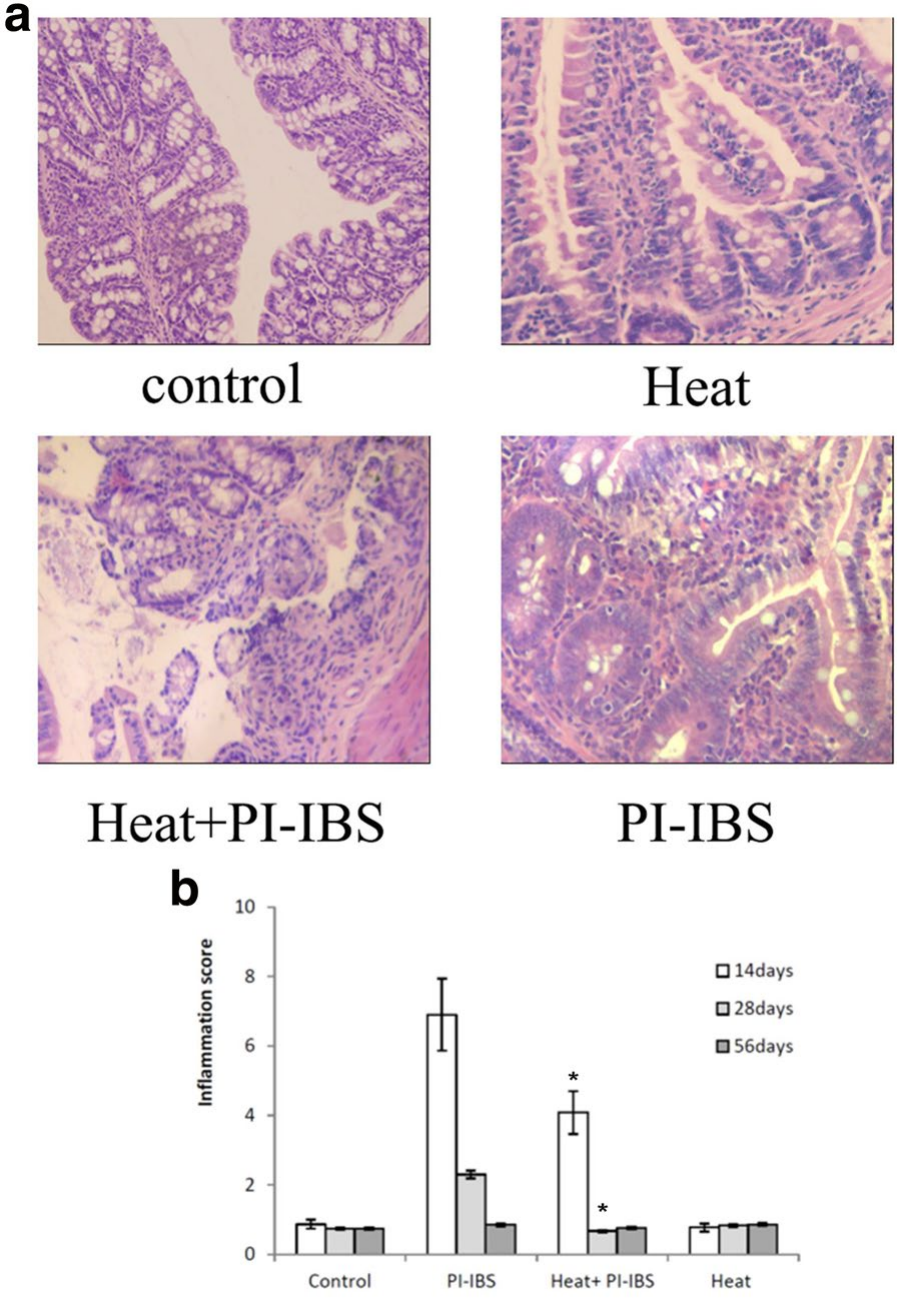
Inflammatory infiltrate in intestine. **a** H&E staining of the ileum; **b** Inflammation score of different groups. *p < 0.05 compared with PI-IBS group

H&E staining of the ileum showed a marked infiltration by *T. spiralis* infection day 14. Infiltration and edema gradually reduced from day 14 until day 56, at which stage no obvious inflammatory infiltrate was observed (Fig. 3a). However, in the heat treated HSP group, the inflammatory infiltrate was significantly reduced compared with HSP group (p < 0.05). Furthermore, the inflammatory score was evaluated. Heat treatment could significantly reduce the inflammatory score compared with HSP group (p < 0.05) (Fig. 3b).

### Hsp70 promoted γδ T cells’ Th17 response

**Fig. 4:**
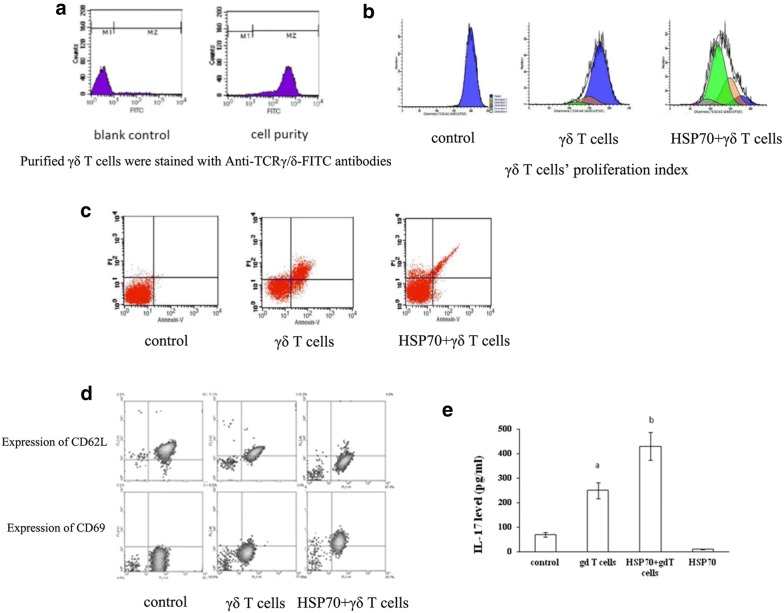
γδ T cells’ Th17 response and IL-17 production by γδ T cells. **a** Purified γδ T cells were stained with Anti-TCRγ/δ-FITC antibodies and runned on FACS; **b** γδ T cells’ proliferation index was measured; **c** Annexin V-FITC/PI apoptosis Kit was used to determine the apoptosis rate; **d** The surface molecular expression of CD62L and CD69 was detected by FACS; **e** The production of IL17 by γδ T cells stimulated by HSP70 was measured by ELISA

After γδ T cells were purified, FITC conjugated anti-TCR mAb and microbeads conjugated anti-FITC mAb staining were used to clear the miscellaneous γδ T cells (Fig. 4a). In the CFSE proliferation assay, the proliferation of γδ T cells were significantly promoted by HSP 70 (Fig. 4b). In the apoptosis assay, HSP 70 could significantly reduce the apoptosis of γδ T cells (Fig. 4c). Moreover, the activation of γδ T cells was also detected. It was shown that the expression of CD62L was significantly reduced by HSP 70, while the expression of CD69 was significantly increased, suggesting that the γδ T cells were activated by HSP 70 (Fig. 4d).

### Effect of HSP70 on the IL-17 production by γδ T cells

IL-17 in the supernatants from the intestinal tissue sample and the culture of γδ T cells were measured by ELISA. It was shown that IL-17 was significantly increased by HPS 70 treatment in γδ T cells (p < 0.05) (Fig. 4e).

## Discussion

The precise mechanism underlying IBS remains unclear, as well as effective therapy.

AWR scores were altered in response to low or medium pressures. When distention volume was 0.25 ml, the pressure was too low to cause any visceral sensation. But when the distention volume was 0.65 ml (high pressure), the level of stimulation was so high that it resulted in a very intense response in both groups of mice. By either 0.35 or 0.5 ml distention volume, the AWR scores in the model group were higher than those in the control group, and the pain threshold in the model group was lower than that in the control group at the same time point. This suggests an increase of the visceral sensitivity in mice after infection. The irritable bowel syndrome (IBS) is a common disorder characterized by abdominal pain in the setting of altered perception of viscerosensory stimuli. Pain is normally evoked by stimuli that are sufficiently intense to activate high-threshold sensory fibers, which relay the signal to the spinal cord. However, tissue injury or inflammation may lead to profoundly increased pain sensitivity in which noxious stimuli generate a greater response (hyperalgesia) and stimuli that are normally innocuous elicit pain (allodynia) [25].

Although the pathogenesis of PI-IBS is not well understood, increasing evidence suggests that low-grade inflammation and immune activation play pivotal roles in the occurrence and persistence of its symptoms. Recently, development in mucosa immunity research provides a potential target for the treatment of IBS.

Our previous research reported that the intestinal HSP70 level raised during PI-IBS. Furthermore, Preinduction of HSP70 could improve the clinical symptoms of PI-IBS [22, 26]. Several studies also demonstrated that HSP70 inhibits the production of proinflammatory cytokines in different cell populations [27]. More recently, Muralidharan et al. reported that association of HSP70 with NF-κB subunit p50 in alcohol-treated macrophages correlates with reduced NF-κB activation and downstream TNF-α, IL-6 and IL-1β production [28]. Intestinal mucosal barrier function is the capacity of the intestine to provide adequate containment of luminal microorganisms and molecules while preserving the ability to absorb nutrients. Alteration of the mucosal barrier function with accompanying increased permeability and/or bacterial translocation has been linked with a variety of conditions, including inflammatory bowel disease [29].

In the current study, we have found that heat treatment could induce the increase expression of intestinal HSP70. It is reported that HSP70 could protect mucosa damage and regulate local inflammation via participating in innate immunity, mediating suppression of the intracellular apoptotic pathway, stabilizing and preventing irreversible aggregation of heat-damaged protein [30–32]. Heat treated PI-IBS mouse show increased number of γδ T cells, suggesting that the proliferation of γδ T cells was increased. Interestingly, it was also observed that IL-17 level was increased in the intestine of PI-IBS mice. These results suggested that HSP70 and γδ T cells maybe simultaneously involved in PI-IBS.

Local low-grade inflammation and immune activation are known as important components of the pathophysiology of PI-IBS. Levels of interferon γ and interleukin (IL)-17 could be significantly increased following *T. spiralis* infection, while IL-10 was decreased [18]. The γδ T cells’ Th17 response plays an important role in many diseases, but the behind mechanism remains unclear. They have also displayed dual effect on the progress of the diseases. Moreover, sometimes they act as a trigger leading to unknown down-stream events [33–35]. It is proved that γδ T cell was not the only one participating in the sophisticated network in the intestinal inflammation and immunity. Some other cells could also produce IL-17 [36–39]. Thus, HSP70 could utilize other pathways during PI-IBS. In our study, γδ T cells were isolated and purified from PI-IBS mouse’s intestine. HSP70 significantly promoted the γδ T cells’ proliferation and activation, prevented their apoptosis, and increased the IL-17 production. These were the potential mechanism how HSP70 exert its protective role in vivo via promotion of γδ T cells’ Th17 response.

There were also some limitations in our study. The type of visceral hypersensitivity in PI-IBS mouse was not investigated. The water content of the bristol stool was not analyzed.

In conclusion, IBS seriously impact the patients’ life quality, which may result from a synergy of multiple etiological factors including the visceral hypersensitivity, the abnormal intestinal motility, local immune response and social psychological factors. Our study has provided a useful clue to explore the pathogenesis of PI-IBS and to search a novel target for this disorder’s therapy in clinical practice. HSP70 plays a protective role via up-regulating intestinal γδ T cell’s Th17 response in PI-IBS mice.

## Abbreviations

HSP70: heat shock protein 70
PI-IBS: post-infectious irritable bowel syndrome
IBS: irritable bowel syndrome
Th: T-helper
IFN: interferon
IL: interleukin
HSP70: heat shock protein 70
HE: hematoxylin–eosin
AWR: abdominal withdrawal reflex
CTT: Colon Transportation Test
RT-PCR: real-time quantitative PCR
